# Fertility preserving management of early endometrial cancer in a patient cohort at the department of women’s health at the university of Tuebingen

**DOI:** 10.1007/s00404-020-05905-8

**Published:** 2021-02-19

**Authors:** Jürgen Andress, Jana Pasternak, Christina Walter, Stefan Kommoss, Bernhard Krämer, Andreas Hartkopf, Sara Yvonne Brucker, Birgitt Schönfisch, Sahra Steinmacher

**Affiliations:** Department of Women’S Health, Tuebingen University Hospital, Calwerstr. 7, 72076 Tuebingen, Germany

**Keywords:** Fertility sparing treatment, Low-grade endometrial cancer, Complex atypical hyperplasia, Progestin agents

## Abstract

**Purpose:**

To investigate the oncologic and reproductive outcome of a conservative treatment with progestin agents in early-stage grade 1 endometrial cancer (G1EC), grade 2 endometrial cancer (G2EC) or complex atypical hyperplasia (CAH) in young premenopausal women.

**Methods:**

Women treated for early-stage endometrial cancer or atypical hyperplasia of the endometrium with a conservative therapy between 2006 and 2018 were enrolled in this retrospective analysis**.** Progestin agents were orally administered on a daily basis for 3 months for at least one cycle. Endometrial tissue was obtained by hysteroscopy and Dilatation & Curettage (D&C) being performed before and after end of treatment. Therapeutic response was assessed by pathological examination.

**Results:**

A total of 14 patients were included. After treatment with progestin agents, 11 of these patients initially showed a complete or partial response. Three patients with early-stage endometrial cancer did not respond.

Of the three patients with initially diagnosed atypical hyperplasia, none showed any remaining disease later. Of the eight patients with initially diagnosed endometrial cancer, who had responded to first treatment, three patients were re-diagnosed with endometrial cancer later. One patient with initial endometrial cancer became pregnant but aborted in the 10th week.

**Conclusion:**

Due to its good efficacy, progestin agents offer a feasible therapeutic option in the fertility-preserving treatment of early-stage endometrial cancer in young premenopausal women. However, recurrence rate remains high. Therefore, a close follow-up is mandatory, also in responders. Patients should be informed of limitations and risks of conservative treatment. Yet after completion of family planning, hysterectomy should be performed.

## Introduction

Endometrial cancer represents the most common entity of gynecologic cancers in Germany and the second most common worldwide. Annually, it is being diagnosed in 11.000 women in Germany and with 4.7% of all new cancer diagnoses, it ranges among the four most common cancer entities behind breast, colorectal and lung cancer (Robert-Koch-Institut). Endometrial cancer typically occurs in postmenopausal women, often detected through vaginal bleeding. As the incidence of causing risk factors, such as diabetes, or adiposity increases in industrialized countries, it is also becoming more prevalent in younger, premenopausal women, accounting for 15–25% of all cases of endometrial cancer [1]. 10% of the patients with endometrial cancer are being diagnosed younger than 45 years, 4% are even under 40 [2].

Among women with genetic predisposition like the Lynch Syndrome, the risk for endometrial cancer rises significantly when it is simultaneously diagnosed with non-insulin-dependent diabetes or hypercholesterinemia [3]. Other risk factors include high blood pressure, early age at menarche and polycystic ovaries [4].

Besides the more frequent occurrences of risk factors in young reproductive woman, a notable number of them are delaying child-bearing, thus leading to an increasing number of nulliparous women being confronted with the diagnosis. An even larger group of women is diagnosed with complex atypical hyperplasia (CAH), the precursor lesion of endometrial cancer [5]. The established recommended surgical procedure in case of endometrial cancer consists of hysterectomy, bilateral salpingo-oophorectomy (BSO) and if necessary depending on tumor stage or histological subtype even of pelvic and paraaortic lymph node assessment (S3-Leitlinie-Endometriumkarzinom). This is commonly not an acceptable approach for women interested in future fertility in spite of the oncologic risk.

Hence, a conservative, fertility-sparing management with the use of progestin agents offers a preferred option for patients being diagnosed with (CAH) or an early stage of endometrial cancer (G1EC) with favorable characteristics, such as the limitation to the endometrium and the good differentiation of the tumor cells. It is assumed that progestin agents induce stromal decidualization and consecutive thinning of the endometrium through activation of progesterone receptors [6].

Previous studies indicate that the progestin treatment provides a feasible option for those patients who wish to preserve fertility by providing sufficient oncologic safety at the same moment [7–10]. The conservative management does not augment the risk of progression of disease or death [11]. Today, established guidelines concerning the application of a standard protocol and treatment duration of progestin agents are still lacking due to the low number of cases. Only few studies have investigated the reproductive outcome due to the short treatment period and follow-up.

In our retrospective analysis, we evaluated the oncologic and reproductive outcome of a conservative management with progestin agents in patients with early-stage endometrial cancer or atypical complex hyperplasia in our department who were seeking a fertility-sparing treatment.

## Materials and methods

Patients with complex atypical endometrial hyperplasia and/or early-stage endometrial cancer being treated conservatively for fertility preservation at the Department of Women’s Health between 2006 and 2018 were included in this retrospective study.

Diagnosis was made through endometrial tissue sampling carried out by hysteroscopy and dilatation and curettage (D&C). In the case of unclear or suspicious histological findings, a re-hysteroscopy and D&C was performed to ensure the diagnosis. Patients were instructed to receive progestin agents orally on a daily basis for 3 months. Clinical follow-ups to evaluate treatment success were equally performed by hysteroscopy and D&C. The therapeutic outcome and treatment response was defined as complete response, partial response, stable disease and progressive disease.

A complete response was defined by no remaining pathological findings after clinical re-evaluation with D&C. Partial response was considered as the change of low-grade endometrial cancer to complex atypical hyperplasia. Stable disease describes no change in histopathological findings. Progressive disease was determined as the change from complex atypical hyperplasia to low-grade endometrial cancer cells. Data extraction was performed with clinical records. The histological criteria according to WHO and to the International Federation of Gynecology and Obstetrics (FIGO) were applied. Approval by the institutional ethics committee of the Medical Faculty of the Eberhard-Karls-University was obtained (299/2017BO2).

Baseline characteristics were described by median, range, mean and standard deviation (SD), respectively, frequencies and proportions. Time to first relapse after last successful treatment was estimated by Kaplan–Meier method. Basic statistics were done by excel version 16.27, for graphics and PFS the software R, version 3.5.1, was used.

## Results

### Patient’s characteristics

Of all 15 patients treated in our department between 2006 and 2018, one patient was excluded due to loss of follow-up. Of the 14 patients included, one patient had been diagnosed with endometrial cancer grading G2 (7%), 10 (71%) had been diagnosed with G1EC, 3 patients (21%) with CAH. The median age of the patients was 32.2 years (range 30.1–47.9 years) at time of diagnosis, with a mean age of 34.2 years (SD 4.8 years). The median body mass index (BMI) was 32.8 kg/m^2^ (range 21.6–47.9 kg/m^2^), the mean BMI was 33.1 kg/m^2^ (SD 8.5 kg/m^2^). Eight patients had a BMI higher than 30 kg/m^2^ (Table 1).

**Table 1:** Baseline characteristics of the 14 patients diagnosed with endometrial cancer or atypical hyperplasia

Initial diagnosis *n* (%)		
Atypical hyperplasia	3	(21%)
Endometrial cancer, G1	10	(71%)
Endometrial cancer, G2	1	(7%)
Age at diagnosis (years) median (range)	32.2	(30.1–47.9)
Body mass index (kg/m^2^) median (range)	32.8	(21.6–47.9)
Body mass index (kg/m^2^) *n* (%)		
< 24.9 kg/m^2^	3	(21%)
25–29.9 kg/m^2^	3	(21%)
30–34.9 kg/m^2^	2	(14%)
35–39.9 kg/m^2^	2	(14%)
≥ 40 kg/m^2^	4	(29%)
Other risk factors *n* (%)		
PCOS	2	(14%)
Diabetes mellitus type II	3	(21%)
Reason for initial diagnosis *n* (%)		
Primary infertility	10	(71%)
Secondary infertility	4	(29%)
Ectopic pregnancy	1	
Caesarian section	2	
Vaginal delivery	1	
Pelvic imaging *n* (%)		
Pelvic ultrasound	14	(100%)
MRI	10	(71%)
Progestin therapy *n* (%)		
MPA 500 mg/day	8	(57%)
MA 160 mg/day	4	(29%)
MA 320 mg/day	1	(7%)
Dydrogesteron 10 mg/day	1	(7%)

*PCOS* Polycystic ovarian syndrome, *MPA* Medroxyprogesterone acetate, *MA* Megestrol acetate, *MRI* Magnetic resonance imaging

Of all 14 patients, 10 (71%) presented with primary infertility, 4 (29%) patients demanded diagnostics due to secondary infertility. Out of the four patients with secondary infertility, two women have had a caesarian section before, one has had an ectopic pregnancy and one had previously delivered vaginally.

### Treatment

The conservative management consisted of the oral application of 500 mg MPA (Medroxyprogesterone acetate), 160 mg MA (Megestrol acetate) or 10 mg Dydrogesteron on a daily basis for 3 months. Eight patients (57%) received MPA, whereas in five patients (35%), MA was administered. One patient (7%) was treated with 10 mg Dydrogesteron. The mean duration of treatment was 4.3 months. The majority of patients received progestin agents for at least 3 months. One patient received for MPA 500 mg for only 1.5 month. Treatment was terminated earlier due to side effects (Table 2).

**Table 2:** Management according to initial diagnosis, age, BMI and treatment duration

Patient ID	Diagnosis	Age at diagnosis (years)	BMI (kg/m^2^)	Therapy (mg)	Treatment duration (months)	Follow-up time (months)	Outcome after first treatment	Outcome after last treatment
1	G2EC	35.9	42.8	Dydrogesteron 10	3	5	SD	/
2	G1EC	30.7	47.9	MA 160	3 + 3 + 3 + 3	33	PR	SD
3	G1EC	37.0	21.6	MPA 500	3 + 3	18	CR	PR
4	G1EC	30.4	38.6	MPA 500	3	5	SD	/
5	G1EC	31.4	42.7	MPA 500	6 + 4	40	PR	CR
6	G1EC	30.2	40.8	MPA 500	3	10	CR	/
7	G1EC	32.9	26.7	MA 160	3	5	SD	/
8	G1EC	35.6	32.2	MA 320	3	6	PR	/
9	G1EC	36.6	26.9	MPA 500	3	4	CR	/
10	G1EC	47.9	33.5	MPA 500	1.5	6	PR	/
11	G1EC	30.6	28.5	MA 160	2.5	40	CR	/
12	CAH	31.6	36.7	MPA 500	3	7	CR	/
13	CAH	30.1	23.4	MA 160	4 + 3	46	CR	CR
14	CAH	37.4	22.2	MPA 500	3	11	CR	/

*G2EC* Endometrial cancer grade 2, *G1EC* Endometrial cancer grade 1, *CAH* Complex atypical hyperplasia, *SD* stable disease, *PR* partial remission, *CR* complete remission

One patient with a BMI of 42.7 kg/m^2^ was treated with MPA 500 mg for 10 months in total, another patient with a BMI of 47.9 kg/m^2^ with MA 160 mg for 12 months in total, both belonging to those three patients with the highest BMI of the cohort. The patient diagnosed with G2EC (BMI 42.8 kg/m^2^) was treated with 10 mg Dydrogesteron for 3 months in total.

Of the ten patients suffering from G1EC, six patients were treated with 500 mg MPA between 1.5 and 10 months in total. Three patients were treated with 160 mg MA, thereof one for 2.5 months, one for three months, one patient for 12 months in total. One patient was treated with 320 mg MA for 3 months. Of the three patients diagnosed with complex atypical hyperplasia, one (BMI 23.4 kg/m^2^) received 160 mg MA for 7 months in total, two were treated with MPA 500 mg for 3 months.

The histological follow-up, based on endometrial tissue samples obtained by hysteroscopy and D&C was performed shortly after treatment, only in one case, the clinical control was performed 5 months after end of treatment, due to non-compliance of the patient. Control-hysteroscopies and D&C were usually performed regularly every three months after end of therapy to ensure treatment success. If no histopathological treatment success could be found, a new treatment cycle was initiated.

## Outcome

### Outcome after first treatment cycle

#### Treatment with MA

In total, four patients of the cohort were treated with MA 160 mg. Three had been diagnosed with G1EC, one patient with CAH. One patient with a G1EC was administered MA 320 mg. After the first cycle of conservative management in the group of three patients with low-grade endometrial carcinoma, who had been treated with 160 mg MA, one patient showed a partial response with remaining CAH. One patient showed complete response.

**Fig. 1:**
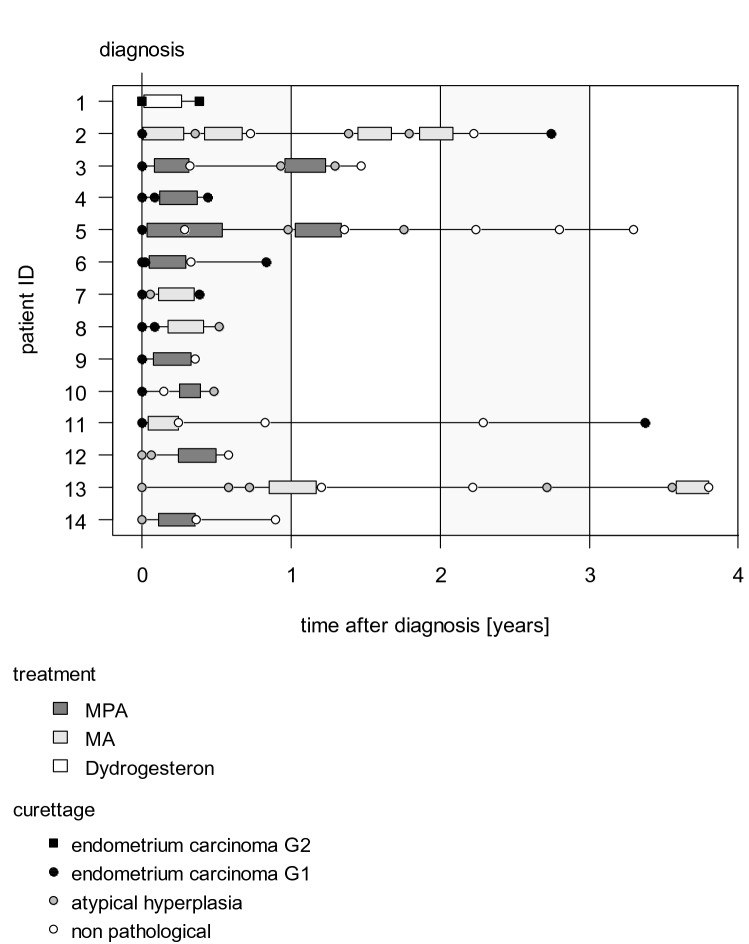
Individual treatment course of each patient demonstrates the individual treatment course of each patient with progestine agents

One patient did not respond to treatment. In the patient treated with 320 mg MA, a partial response with remaining CAH could be seen. The patient with the initial diagnosis of atypical hyperplasia and MA treatment showed a complete remission after the first cycle of treatment (see Fig. 1).

#### Treatment with MPA

Eight patients were treated with 500 mg MPA, thereof six with low-grade endometrial cancer, two with atypical hyperplasia. Three patients with endometrial carcinoma had a complete response with no remaining disease after one cycle of treatment of three months. Two patients showed partial remission with remaining atypical hyperplasia. One of those had been treated for only one month, as treatment had been finished earlier due to side effects.

One patient did not respond to MPA therapy and showed remaining cancer cells. This patient underwent surgical treatment with total hysterectomy and BSO. The two patients with initially diagnosed CAH showed a complete response after one cycle of treatment.

#### Treatment with Dydrogesteron

One patient diagnosed with G2EC received Dydrogesteron 10 mg for 3 months but did not respond.

### Outcome after various treatment cycles

Four patients were treated for more than one cycle with progestin agents. One patient with G1EC (ID 2) was treated with four cycles of MA 160 mg in total. After the first cycle, partial remission with remaining complex atypical hyperplasia was obtained. The patient then was treated with a second cycle with MA. In the following D&C, a complete remission was diagnosed. Yet in the following control D&Cs, the patient relapsed with treatment control revealed, still remaining complex atypical hyperplasia. Thus, the patient received a fourth cycle of MA. After treatment, complete remission was seen. In the subsequent curettage 8 months after end of the last treatment, the patient was re-diagnosed with endometrial cancer.

Another patient with G1EC (ID 3) showed complete remission after 3 months of treatment with 500 mg MPA. She then relapsed with CAH, so another treatment cycle with 500 mg MPA was administered. The control D&C performed directly after the end of the second treatment cycle revealed remaining CAH. In the control hysteroscopy and D&C after 3 months, complete remission could be diagnosed again.

A third patient with G1EC (ID 5) was treated with MPA 500 mg for two cycles. During the first treatment cycle, complete remission could be seen. In the control D&C, the patient relapsed with complex atypical hyperplasia. Hence, a second treatment cycle was started. In the first histopathological control, after the second treatment cycle, no pathology was found. The following curettage again revealed complex atypical hyperplasia. The patient then had two control D&Cs, both with no pathological findings.

One patient initially diagnosed with CAH (ID 13) also received two cycles of treatment with MA 160 mg. After the first treatment cycle, complete remission was diagnosed. Yet the patient relapsed with CAH and was treated with another cycle of MA. In the control D&C, complete remission was again diagnosed (see Fig. 1).

For the nine patients with response to therapy, median duration to first progression after last successful treatment was 7.8 months.

Of all 14 patients, one patient with initial endometrial cancer became pregnant but aborted in the 10th week.

## Discussion

In this retrospective study, we analyzed the outcome of 14 patients with CAH, G1EC and G2EC being treated by different progestin agents. A treatment response after the first therapy cycle could be noticed in 11 patients, three with low-grade complex atypical hyperplasia, and eight with endometrial cancer. Yet, in three of these eight patients, the endometrial cancer was re-diagnosed after conservative progestin therapy. At the end of follow-up, two patients with an initially diagnosed endometrial cancer showed atypical endometrial hyperplasia. Unfortunately, none of our patients achieved a successful pregnancy, which was the primary goal of the conservative treatment regime. One patient with initial endometrial cancer became pregnant but aborted in the 10th week. The results highlight both, the success and limitations of the treatment with progestin agents in young women wanting to retain future fertility potential.

Until today, standardized therapy regimen concerning the fertility-preserving treatment of endometrial neoplasia is lacking. Numerous previous investigations have shown the effects of progestin agents in the fertility-preserving treatment of young patients with complex atypical hyperplasia or low-grade G1EC, whereas fertility-sparing treatment options in Stage IA and G2EC have been investigated only by few studies [12, 13]. According to Thipgen et al., the response rate of progestin agents varies between 37% for G1, 23% for G2 and 9% for G3, assuming that grading is one of the most influential parameters in the outcome after progestin therapy [14].

Ohyagi-Hara et al. treated 27 patients with 400–600 mg MPA daily and demonstrated an initial complete response of 81% in patients with CAH and of 69% in patients treated for G1EC. None of the patients with CAH recurred, however, 81% of the patients with G1EC [15]. In a cohort of 59 patients, Fujiwara et al. reported an initial complete response of 71% in patients with G1EC after treatment with MPA, 52% of the patients relapsed over time [16]. In accordance to the systematic review of Gunderson et al., we also noted higher recurrence rates in patients with endometrial carcinoma [6] than in those with complex atypical hyperplasia [6]. This emphasizes the need for an immediate start of fertility treatment after achieving complete remission.

All patients of our study initially presented due to infertility problems, the uterine neoplasia was diagnosed accidently. Considering one of the primary goals of the treatment, the fertility preservation, it remains questionable whether the delay of a therapy with sufficient oncologic safety is justified by the doubtful perspective of a successful pregnancy in our cohort. This therapeutic dilemma is even underlined by the fact that the standard surgical procedure of total hysterectomy and BSO has a very good prognosis in premenopausal women [17].

To date, defined therapeutic regimens and application methods, as well as data concerning dosage and duration of progestin treatment are lacking. New approaches to establish more effective treatment regimen have been performed recently. In a systematic review, Gallos et al. demonstrated that Levenorgestrel-IUD (LNG-IUD) had a higher regression rate in atypical hyperplasia than the use of oral progestins. This therapeutic advantage did not apply for G1EC [18]. Kim et al. evaluated the therapeutic effect of a combination of oral progestins and LNG-IUD in 16 patients with G1EC. Here, 87% of all patients had an initial complete response. Of those, 14% recurred. This might suggest that a combination of both can be more effective than an oral application. The use of additional parameters that may predict treatment success can also be helpful to evaluate an individual therapeutic regimen.

In recent studies, potential molecular markers for the prediction of the possibility of achieving complete remission in early-stage endometrial cancer have been identified. Yamazawa et. al demonstrated that the positive expression of progesterone receptor was related to the rate of complete remission of low-grade endometrial cancer after MPA therapy [19]. Yet, therapy with progestin agents has proven to be effective in the treatment of hormone-negative tumors, suggesting there might be another effect of progestin on tumor cells than through the mediation of hormonal receptors [20]. The expression of the cell adhesion molecule L1-CAM in the presence of an endometrial carcinoma stage 1 may also give additional information regarding the tumor biology and prognosis. Kommoss et al. recently reported that an overexpression of L1CAM is associated with a more aggressive tumor entity and potential distant metastasis in the event of recurrence [21]. This marker might play an influential role in the future treatment algorithm of early-stage endometrial carcinoma.

The results of our study have to be interpreted in the light of some limitations. First, the study is of retrospective nature at a single institution and reveals a small sample size. Moreover, different progestin agents were used and no defined treatment duration was applied. This is due to the individualized treatment course of each patient, as therapeutic success was frequently evaluated and changes in therapy were applied.

## Conclusion

Progestin agents are an effective treatment option in young patients with complex atypical hyperplasia or early-stage endometrial cancer who desire fertility-preserving therapy. Even after sufficient success of conservative therapy close clinical follow-up is mandatory and surgical procedure should be performed with no delay. Patients should be informed about limitations and possible risks of a conservative treatment compared to a definite surgical procedure. Fertility treatment should be taken into consideration after complete remission of CAH or early-stage endometrial cancer, as risk factors that favour the occurrence of endometrial neoplasia on the one hand and reduce fertility on the other hand are often prevalent in this patient group.

Hence, prospective studies with a greater study population and the identification of factors that allow a prediction of the therapeutical response to progestins are highly demanded to establish a reliable and individualized therapeutic regimen.

## Data Availability

Available upon request.
